# Functional Traits Resolve Mechanisms Governing the Assembly and Distribution of Nitrogen-Cycling Microbial Communities in the Global Ocean

**DOI:** 10.1128/mbio.03832-21

**Published:** 2022-03-14

**Authors:** Wen Song, Jihua Liu, Wei Qin, Jun Huang, Xiaoli Yu, Mengzhao Xu, David Stahl, Nianzhi Jiao, Jizhong Zhou, Qichao Tu

**Affiliations:** a Institute of Marine Science and Technology, Shandong University, Qingdao, China; b Joint Lab for Ocean Research and Education at Dalhousie University, Shandong University and Xiamen University, Qingdao, China; c Department of Microbiology and Plant Biology, University of Oklahoma, Norman, Oklahoma, USA; d Environmental Microbiomics Research Center, School of Environmental Science and Engineering, Southern Marine Science and Engineering Guangdong Laboratory (Zhuhai), Sun Yat-sen University, Guangzhou, China; e Department of Civil and Environmental Engineering, University of Washington, Seattle, Washington, USA; f Institute of Marine Microbes and Ecospheres, Xiamen University, Xiamen, China; g Earth and Environmental Sciences, Lawrence Berkeley National Laboratory, Berkeley, California, USA; University of California, Irvine

**Keywords:** marine nitrogen cycle, functional traits, diversity patterns, community assembly, stochasticity, functional redundancy

## Abstract

Microorganisms drive much of the marine nitrogen (N) cycle, which jointly controls the primary production in the global ocean. However, our understanding of the microbial communities driving the global ocean N cycle remains fragmented. Focusing on “who is doing what, where, and how?”, this study draws a clear picture describing the global biogeography of marine N-cycling microbial communities by utilizing the *Tara* Oceans shotgun metagenomes. The marine N-cycling communities are highly variable taxonomically but relatively even at the functional trait level, showing clear functional redundancy properties. The functional traits and taxonomic groups are shaped by the same set of geo-environmental factors, among which, depth is the major factor impacting marine N-cycling communities, differentiating mesopelagic from epipelagic communities. Latitudinal diversity gradients and distance-decay relationships are observed for taxonomic groups, but rarely or weakly for functional traits. The composition of functional traits is strongly deterministic as revealed by null model analysis, while a higher degree of stochasticity is observed for taxonomic composition. Integrating multiple lines of evidence, in addition to drawing a biogeographic picture of marine N-cycling communities, this study also demonstrated an essential microbial ecological theory—determinism governs the assembly of microbial communities performing essential biogeochemical processes; the environment selects functional traits rather than taxonomic groups; functional redundancy underlies stochastic taxonomic community assembly.

## INTRODUCTION

The global ocean is the largest reservoir of reactive nitrogen (N) on Earth and contains five times more biologically available N than the terrestrial ecosystem (1). As an essential element and limiting nutrient, the cycling of marine N jointly controls the ocean productivity, supports marine ecosystems, and facilitates interactions among different organisms (1–4). Similar to other element cycles, the marine N cycle is also mainly driven by microbial communities, via eight main pathways/processes, including nitrification, denitrification, assimilatory nitrate reduction, dissimilatory nitrate reduction, nitrogen fixation, anammox and organic nitrogen metabolism (a detailed illustration of the N-cycling pathways is also available in Fig. 5, below) (2, 5). In addition to the earlier discovery of anammox (6, 7), during the past few years, additional novel discoveries have been made and have greatly expanded our knowledge about this critical biogeochemical cycle. These include but are not limited to novel N_2_-fixing marine microorganisms (4, 8), widespread distribution of ammonia-oxidizing *Thaumarchaeota* in the ocean (9–11), and the discovery of comammox microorganisms (12, 13). However, our understanding about the diversity patterns of microbial communities in the global ocean remains surprisingly poor (14), including the microbial communities mediating the marine N cycle.

Characterizing the biogeography and diversity patterns of microbial communities mediating the marine N cycle and the associated geo-environmental drivers in the global ocean is of critical importance to unraveling the ecology of N-cycling communities and further predicting how global environmental changes will alter the marine N cycle and vice versa (15–18). Microbial functional traits have recently been integrated with ocean biogeography and biogeochemistry models (19). Previous studies have suggested clear latitudinal gradient patterns for marine plankton, with temperature as the main environmental driver for such patterns (14, 20–22), though some exceptions have also been observed (23, 24). Besides temperature, factors including oxygen, chlorophyll *a*, ocean primary production, pH, and salinity are also reported to be important environmental parameters shaping the biogeography of marine microbial communities (18, 20, 21, 23). However, identifying the geo-environmental factors shaping the marine N-cycling communities at the global scale has remained elusive.

The large volume of metagenomic data generated by the *Tara* Oceans expedition (20) allows us to now comprehensively relate the functional traits mediating different steps in the marine N cycle to the distribution of those traits among different microbial taxa and associated geo-environmental factors. In this study, we aimed to address a set of fundamental questions to advance our understanding of the microbial communities driving the global marine N cycle. (i) How are N-cycling functional traits and taxonomic groups distributed in the global ocean? Previous studies suggested that either in the human gut or natural ecosystems, essential ecosystem functions are maintained by a set of core functional genes/traits (25–27). We therefore expected that the composition of N-cycling functional traits would be relatively even globally, while their taxonomic composition may vary dramatically. (ii) Do N-cycling functional traits and taxonomic groups follow any biogeographic patterns such as latitudinal diversity gradients (LDG) and distance-decay relationships (DDR)? We expected such patterns for N-cycling taxonomic groups, but the patterns should be much weaker or even not exist for functional traits, as functional traits should be relatively stably distributed in the environment to maintain essential ecosystem functions. (iii) What geo-environmental factors shape observed diversity patterns? The marine N cycle is coupled with many other element cycles, in particular, carbon and phosphorus (1, 2). Besides temperature and oxygen (20), we also expected biologically available nitrogen and phosphorus to have big impacts on the marine N cycle. (iv) What roles do deterministic and stochastic assembly processes have in structuring N-cycling microbial communities? As an essential nutrient cycle, we expected the marine N cycle to be under strong selection pressure by the ecosystem (i.e., deterministic assembly), especially the functional traits. However, owing to functional redundancy of microbial communities (25), taxonomic groups would be subject to a higher degree of stochasticity than functional traits.

## RESULTS AND DISCUSSION

### How are N-cycling functional traits and taxonomic groups distributed in the global ocean?

**FIG 1:**
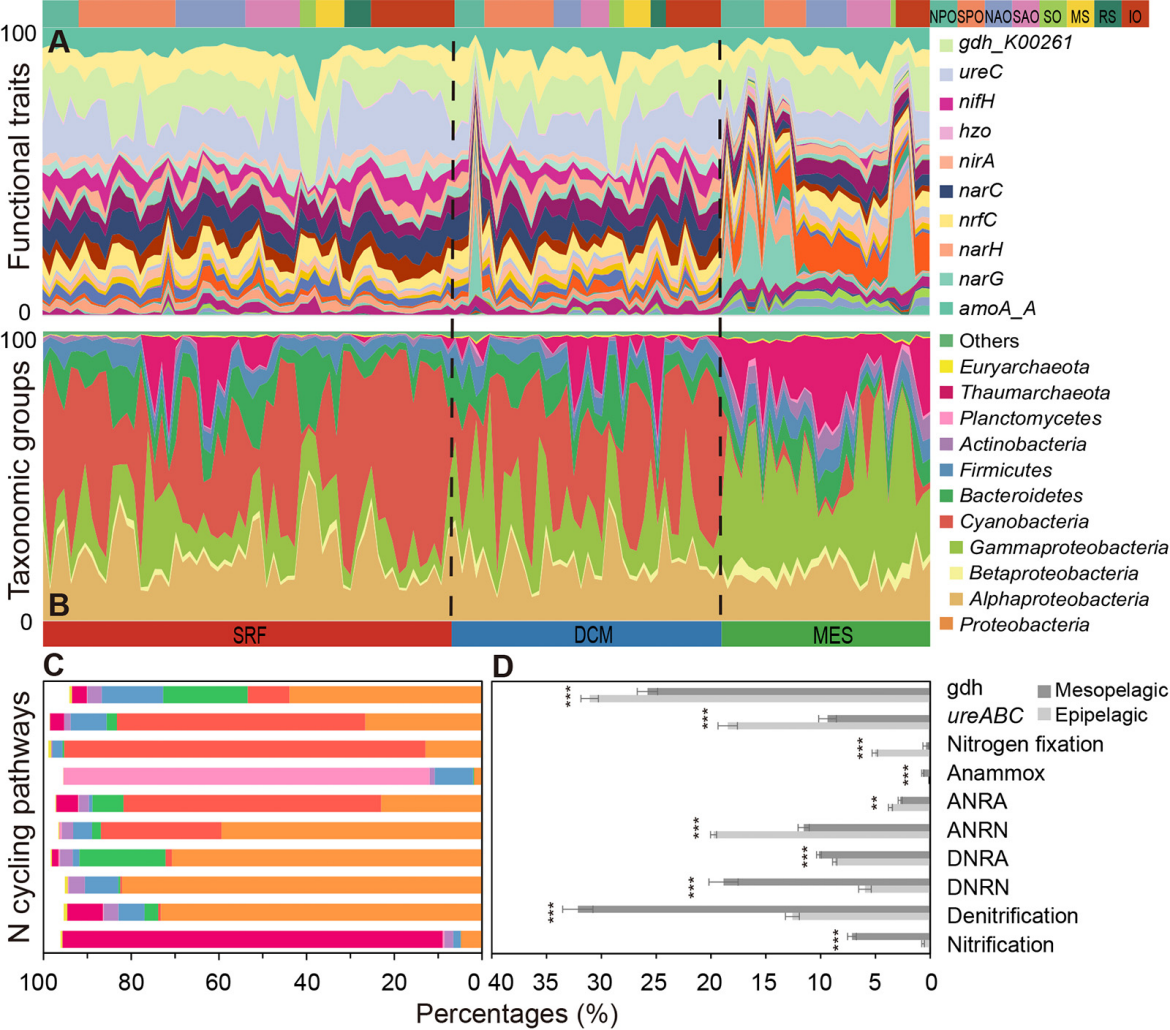
The composition of N-cycling functional traits and taxonomic groups in the global ocean as revealed by the *Tara* Oceans metagenomes. (A) The relative abundance of microbial functional traits in different oceans and layers. Only representative gene families with high relative abundances were annotated in the figure. The exact relative abundance for each gene family can be found in Table S2. (B) The relative abundance of microbial phyla in different oceans and pelagic zones. Here, *Proteobacteria* was further divided into *Alpha*-, *Beta*-, and *Gammaproteobacteria*. (C) The composition of microbial phyla mediating different N-cycling pathways. Here, the same color code as in panel B was used. (D) The relative abundance of N-cycling pathways in epipelagic and mesopelagic zones. Significant differences between epipelagic and mesopelagic are marked with asterisks (**, *P < *0.01; ***, *P < *0.001). NPO, North Pacific Ocean; SPO, South Pacific Ocean; NAO, North Atlantic Ocean; SAO, South Atlantic Ocean, SO, Southern Ocean; MS, Mediterranean Sea; RS, Red Sea; IO, Indian Ocean; SRF, surface water layer; DCM, deep chlorophyll maximum layer; MES, mesopelagic zone. DNRN, dissimilatory nitrate reduction to nitrite; DNRA, dissimilatory nitrite reduction to ammonia; ANRN, assimilatory nitrate reduction to nitrite; ANRA, assimilatory nitrite reduction to ammonia.

Different compositional patterns were observed for functional traits and taxonomic groups. The overall relative abundance of different functional traits was relatively even across different samples, though a few exceptions were observed (Fig. 1A). In contrast, their taxonomic composition varied dramatically, even at the phylum level (Fig. 1B), showing clear functional redundancy scenarios (25). *Proteobacteria* (17.3 to ∼82% relative abundance) dominated the marine N cycle across all *Tara* Oceans samples. Consistent with many other observations, *Cyanobacteria* were abundant in the epipelagic zone (surface water and deep chlorophyll maximum layers; here, SRF and DCM) samples, but not in the mesopelagic layer (here, MES) samples (Fig. 1B). Also consistent with past observations of soil (28) and marine systems (2), certain processes showed a high degree of specialization by taxonomic group (Fig. 1C) (28). For instance, functional traits for nitrogen fixation, anammox, and ammonia oxidation are mainly mediated by *Cyanobacteria*, *Planctomycetes*, and *Thaumarchaeota*, respectively, in marine systems (4, 9, 29, 30).

Both the composition of functional traits and taxonomic groups differed dramatically between epipelagic and mesopelagic zone samples, but not between different oceans (Fig. S1, Table S1). Different N-cycling pathways localize to the epipelagic and mesopelagic regions of the water column. The relative abundance of key functional traits involved in nitrification, denitrification, dissimilatory nitrate reduction to nitrite (here, DNRN), dissimilatory nitrite reduction to ammonia (here, DNRA), and anammox was significantly higher in MES than in epipelagic samples, while those involved in N_2_ fixation, assimilatory nitrate reduction to nitrite (here, ANRN), assimilatory nitrite reduction to ammonia (here, ANRA), and ammonification that produces ammonia via organic decomposition (e.g., *ureABC* and *gdh*) were significantly lower (Fig. 1D, Table S2). Archaeal *amo* gene families, representing functional traits converting NH_4_^+^ to NH_2_OH, were dominant. The archaea to bacteria ratio for *amoA* was ∼29.5 in the epipelagic layer and ∼355 in the mesopelagic layer (Table S2, Fig. S2A). This was consistent with previous studies (10, 31, 32) and suggested that archaea rather than bacteria are mainly responsible for ammonia oxidation in deep ocean layers. A much higher abundance of *nxr* gene families was also observed in mesopelagic layers (Fig. S2B), indicating potential interactive interdomain relationships between ammonia-oxidizing archaea and nitrite-oxidizing bacteria (33). The relative abundance of the key gene family *nosZ*, which represents a functional trait that converts N_2_O to N_2_, did not differ significantly between epipelagic and mesopelagic layers, confirming that N_2_O consumption takes place both in and above oxygen-deficient zones (34). However, *nirS* and *nirK*, gene families responsible for nitrite reduction to nitric oxide, were abundant and differed dramatically by layers. Of these, *nirK* was more abundant in the mesopelagic than in the epipelagic layer, while *nirS* showed the opposite pattern. Such different patterns between *nirK* and *nirS* could be due to the different availability of iron and copper in different layers (35) and the potential competition for iron by phototrophs in the epipelagic layer (36). Taxonomic composition mediating different N-cycling pathways was generally more similar between SRF and DCM layers, but those mediating anammox were more similar between DCM and MES layers (Fig. S3). In fact, anammox gene families were mainly found in oxygen minimum zones (Fig. S2C). Such results suggested that marine N-cycling communities were subject to clear niche differentiation (37) at both the taxonomic and functional trait levels.

10.1128/mbio.03832-21.1 FIG S1 (A and B) Principal-component analysis of the composition of N-cycling taxonomic groups (A) and functional traits (B). Bray-Curtis dissimilarity was calculated to generate the distance matrix. Different colors indicate samples from different oceans, whereas different shapes indicate samples in different layers. Download FIG S1, TIF file, 0.3 MB. Copyright © 2022 Song et al. 2022 Song et al. https://creativecommons.org/licenses/by/4.0/ This content is distributed under the terms of the Creative Commons Attribution 4.0 International license.

10.1128/mbio.03832-21.2 FIG S2 (A to C) Relative abundances of functional traits responsible for ammonification (A), *nxr* gene families (B), and anammox (C) in different pelagic zones. Archaea dominated ammonification in the ocean, no matter which layer was analyzed. Download FIG S2, TIF file, 0.9 MB. Copyright © 2022 Song et al. 2022 Song et al. https://creativecommons.org/licenses/by/4.0/ This content is distributed under the terms of the Creative Commons Attribution 4.0 International license.

10.1128/mbio.03832-21.3 FIG S3 (A to C) The composition of microbial phyla involved in different N-cycling pathways in surface water (A), deep chlorophyll maximum layer (B), and mesopelagic layer (C). Microbial taxa for different N-cycling pathways were generally more similar between SRF and DCM layers than that in MES layer, except annamox. Download FIG S3, TIF file, 0.8 MB. Copyright © 2022 Song et al. 2022 Song et al. https://creativecommons.org/licenses/by/4.0/ This content is distributed under the terms of the Creative Commons Attribution 4.0 International license.

10.1128/mbio.03832-21.8 TABLE S1 Statistical analysis of the compositional differences between epipelagic (MES and DCM) and mesopelagic (MES) layers. Three nonparametric statistical approaches, including permutational multivariate analysis of variance (PERMANOVA), analysis of similarity (ANOSIM), and multiple response permutation procedure (MRPP), were carried out based on Bray-Curtis dissimilarity distance matrices. Both compositions for taxonomic groups and functional traits were analyzed. Download Table S1, DOCX file, 0.01 MB. Copyright © 2022 Song et al. 2022 Song et al. https://creativecommons.org/licenses/by/4.0/ This content is distributed under the terms of the Creative Commons Attribution 4.0 International license.

10.1128/mbio.03832-21.9 TABLE S2 Relative abundance of microbial functional traits involved in N-cycling processes across all ocean samples. Download Table S2, XLSX file, 0.1 MB. Copyright © 2022 Song et al. 2022 Song et al. https://creativecommons.org/licenses/by/4.0/ This content is distributed under the terms of the Creative Commons Attribution 4.0 International license.

### Do N-cycling communities follow LDG and DDR patterns?

Two types of biogeographic diversity patterns, including LDG and DDR, were examined for N-cycling functional traits and taxonomic groups. Although the distribution of functional traits is routinely studied in macroecology and characterizing the biogeography of microbial functional traits is of recognized importance (17), there are relatively few examples of this analysis applied to microorganisms (28, 38, 39). Here, considering the essential ecosystem function they perform, we expected N-cycling functional traits to be prevalently and relatively stably distributed in the ocean. Biogeographic patterns for N-cycling functional traits were therefore expected to be much weaker than that for taxonomic groups, or even to not exist.

**FIG 2:**
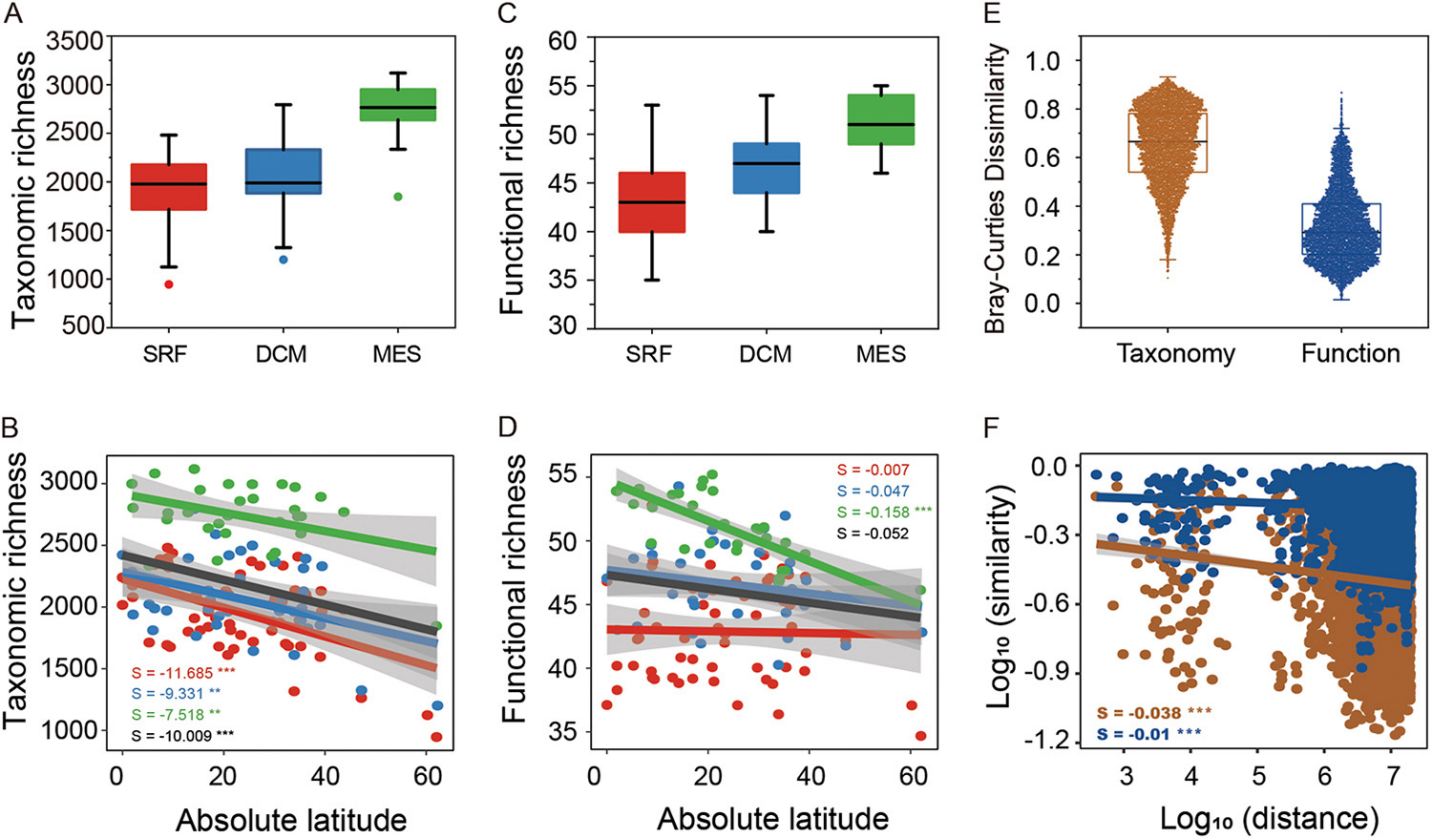
Biogeographic diversity patterns of N-cycling taxonomic groups and functional traits in the ocean. The vertical and latitudinal diversity patterns of taxonomic richness (AB) and functional trait richness (CD), as well as the community dissimilarity and the distance-decay relationship of taxonomic groups and functional traits (EF) were investigated. The black line in panels B and D represents the latitudinal diversity pattern for N-cycling communities across the whole upper ocean. The observed number of microbial species and gene families involved in N cycling is used here for richness. Statistical significance was indicated with asterisks (**, *P < *0.01; ***, *P < *0.001).

**LDG.** LDG describes the pattern in which species richness decreases with increasing latitude (40). It is a well-recognized pattern for both plants and animals (16, 40) and has also been observed for planktonic marine bacteria and terrestrial microbes (21, 41). This pattern, however, was not clearly observed in the *Tara* Oceans metagenomics data analysis, in which species richness peaks at the midlatitude (20). Consistent with the *Tara* Oceans observations (20), both the N-cycling taxonomic group and functional trait richness increased with depth (Fig. 2A and C). Inconsistently, our analysis did reveal strong LDG patterns for N-cycling taxonomic groups in all samples and samples in different layers (Fig. 2B). For functional traits, this pattern was only observed in the MES layer, but not globally or in epipelagic layers (Fig. 2D). Thus, our study has emphasized the importance of incorporating depth-resolved patterns of diversity in an analysis of latitudinal gradients.

**DDR.** DDR, in which community similarity decreases as geographic distance increases, is another fundamental pattern in ecology (42, 43). DDR is well recognized for microbial communities (44–46) and has also been observed for the entire microbial community in the *Tara* Oceans database (20). Consistent with our expectation, steeper DDR patterns were observed for N-cycling taxonomic groups (*S* = −0.04, *P < *0.001) than for functional traits (*S* = −0.01, *P < *0.001), on the basis that taxonomic composition was more dissimilar than functional trait composition (Fig. 2E and F, Fig. S4A and B). Notably, the slopes for taxonomic DDRs were similarly steep in different layers (*S* = −0.11 to ∼−0.09, *P < *0.001) (Fig. S4A). However, the slopes for functional trait DDRs were only steep in the MES layer (*S* = −0.07, *P < *0.001), but relatively flat in the SRF and DCM layers (*S* = −0.04 and −0.03, *P* < 0.01) (Fig. S4A). Such distinct spatial patterns between taxonomic groups and functional traits have also been observed in freshwater ponds, though different techniques have been used to measure functional traits (47). Taking the LDG pattern, the results suggested that N-cycling communities in the MES layer might have been subject to stronger environmental selection and weaker dispersal (43).

10.1128/mbio.03832-21.4 FIG S4 (A and B) Distance-decay relationship (DDR) (A) and community dissimilarity (B) for N-cycling microbial communities in different ocean layers. Both the community similarity for taxonomic groups at the species level and functional traits were analyzed. Stronger DDRs were observed for taxonomic groups than functional traits. The Bray-Curtis dissimilarity was employed to calculate community dissimilarity. Different colors indicate different ocean layers. Download FIG S4, TIF file, 1.8 MB. Copyright © 2022 Song et al. 2022 Song et al. https://creativecommons.org/licenses/by/4.0/ This content is distributed under the terms of the Creative Commons Attribution 4.0 International license.

### What geo-environmental factors drive marine N-cycling community diversity patterns?

**FIG 3:**
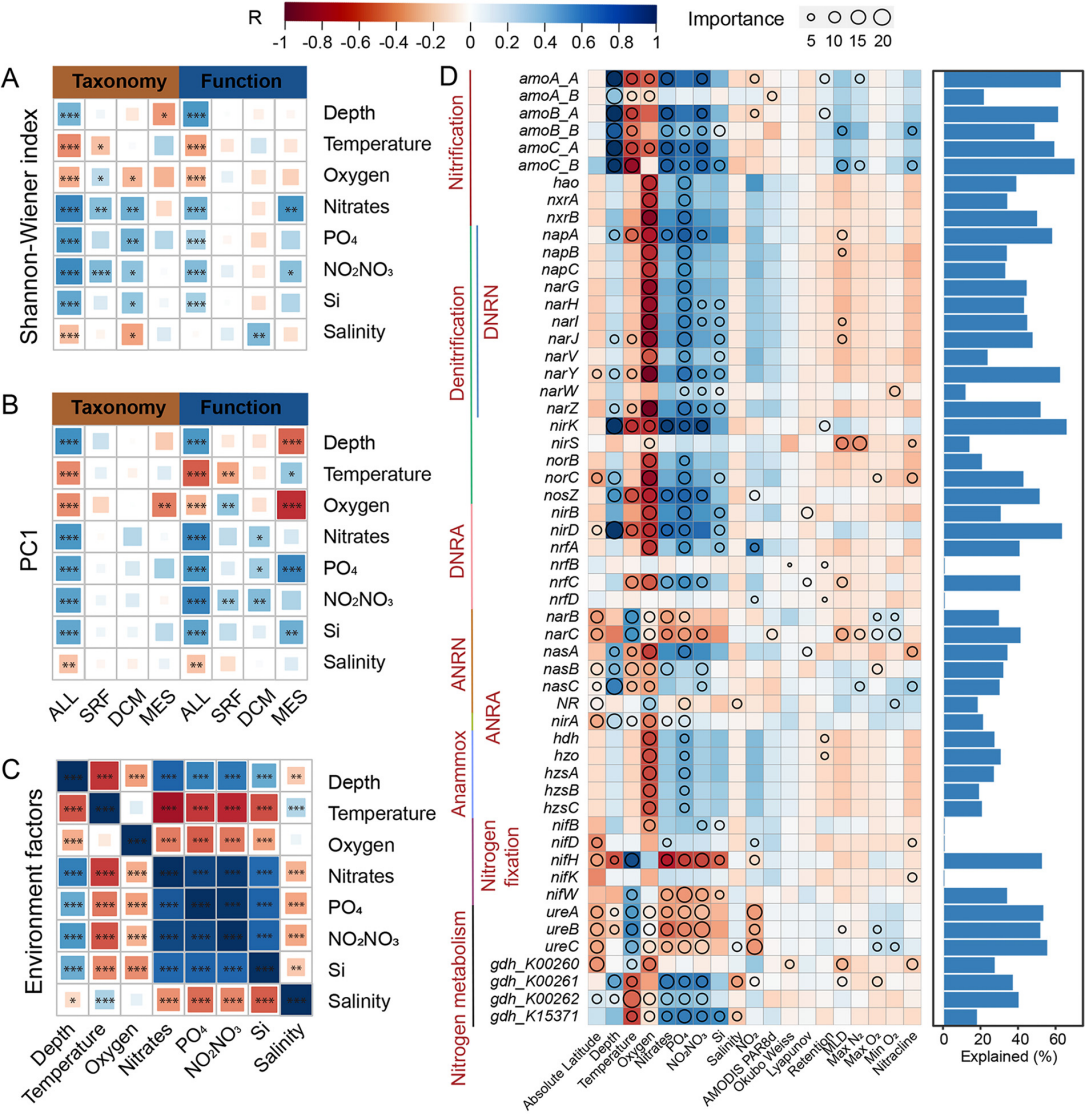
The importance of geo-environmental factors explaining the variations of N-cycling community diversity and composition. (A) Associations between geo-environmental factors and community diversity (Shannon-Wiener index). (B) Associations between geo-environmental factors and community composition (first axis of principal-component analysis). (C) Relationship between different geo-environmental factors (upper right, Pearson correlation coefficient; lower left: Spearman’s rho). (D) Variations explainable by different geo-environmental factors at functional trait level; in the left panel, the associations between the relative abundance of individual functional traits and geo-environmental factors are indicated by the heatmap, whereas the importance of geo-environmental factors in explaining the variations of individual functional traits by random forest analysis is indicated by different sizes of circles; in the right panel, variations explained by the best geo-environmental factor (the one with the largest circle) are indicated by bar plots. DNRN, dissimilatory nitrate reduction to nitrite; DNRA, dissimilatory nitrite reduction to ammonia; ANRN, assimilatory nitrate reduction to nitrite; ANRA, assimilatory nitrite reduction to ammonia. For panels A to C, significance levels for association analyses are marked with asterisks (*, *P < *0.05; **, *P < *0.01; ***, *P < *0.001). For panels A to D, the same scaling color bar was used.

Multiple statistical tests, including linear regression analysis, partial Mantel tests, and random forest modeling, were carried out to disentangle the importance of geo-environmental factors behind the variations of N-cycling community diversity and composition. Besides factors such as temperature and oxygen that drive the whole *Tara* Oceans microbiome (20), multiple geo-environmental factors such as depth, salinity, silicate, nitrate, phosphorus, and NO_2_NO_3_ (NO_2_^–^+NO_3_^–^), were also strongly associated with the overall diversity and compositional variations of marine N-cycling communities (Fig. 3A and B, Fig. S5). Among these, factors such as depth, salinity, and silicate have been previously noted to influence ocean microbiomes (18, 21, 48). Notably, in the soil ecosystem, environmental drivers were only identified for N-cycling functional traits, but not for taxonomic composition (28). Here in the ocean ecosystem, the N-cycling taxonomic groups and functional traits were shaped by the same set of geo-environmental factors, including depth, temperature, oxygen, NO_3_^–^, PO_4_^–^, NO_2_NO_3_, Si, and salinity (Fig. S5), suggesting that the responses of marine N-cycling functional traits and taxonomic groups to geo-environmental conditions were coupled. This is in variance with a soil N cycle study (28), as well as another study in the ocean, together showing that environmental factors strongly influence marine microbiome functional groups but weakly influence taxonomic composition within the groups (49).

10.1128/mbio.03832-21.5 FIG S5 The association of geo-environmental factors and N-cycling microbial communities. Both the compositional variations for taxonomic groups and functional traits were analyzed. The width and color of lines, respectively, indicate the values of Mantel’s *r* and *P* values. Colored circles among different geo-environmental factors indicate Spearman’s rho between different factors. Download FIG S5, TIF file, 1.0 MB. Copyright © 2022 Song et al. 2022 Song et al. https://creativecommons.org/licenses/by/4.0/ This content is distributed under the terms of the Creative Commons Attribution 4.0 International license.

Among these geo-environmental factors, depth was the most influencing factor, differentiating MES from epipelagic layer samples both taxonomically and functionally (Fig. 1A and B, Fig. 3, Fig. S1, Table 1). Weakened correlations with the rest of the geo-environmental factors were observed for samples recovered from individual layers, i.e., when depth effect was removed, confirming the importance of depth (Fig. 3A and B). Similar to other ocean studies, since some of the geo-environmental factors were strongly correlated with each other, it was therefore statistically difficult to identify the “most” important one (Fig. 3C, Fig. S5). Temperature (Spearman’s ρ = −0.47) and oxygen (ρ = −0.36 and −0.46) were negatively associated with the overall N-cycling community diversity and composition, while nitrate (ρ = 0.65 and 0.58), NO_2_NO_3_ (ρ = 0.62 and 0.54), phosphorus (ρ = 0.57 and 0.54), and silicate (ρ = 0.52) were positively associated (Fig. 3A and B, Fig. S5). This suggested that the overall marine N-cycling communities favored lower-temperature and lower-oxygen environments (50), though individual pathways may differ. Strong associations of N-cycling community diversity with nitrate and NO_2_NO_3_ were observed, but not for the entire ocean microbiome (20). This suggested that nitrate, which sources from the deep ocean reservoir and/or terrestrial runoff (51), could be an important driver for the marine N-cycling communities. The strong association with phosphorus also suggested that marine N and phosphorus cycles may also be highly coupled (1, 2, 39).

Geo-environmental factors influencing the whole N-cycling community were also strongly associated with individual functional traits. Functional traits catalyzing the same pathway were generally driven by the same geo-environmental factors (Fig. 3D). For example, all functional traits (*hdh*, *hzo*, and *hzsABC*) involved in anammox were mainly influenced by oxygen and PO_4_^–^. The *amo* gene family, representing the functional trait for converting NH_4_^+^ to NH_2_OH, were all mainly driven by depth and temperature, and functional traits (*hao*, *nxrAB*) that oxidize NH_2_OH to NO_3_^–^ were mainly driven by oxygen. Taxonomically, oxygen was the factor strongly influencing most taxonomic groups, including the dominant *Proteobacteria* and *Thaumarchaeota* (Fig. S6), and depth was strongly associated with *Thaumarchaeota*, *Actinobacteria*, *Verrucomicrobia*, *Chloroflexi*, *Chlorobi*, and *Acidobacteria* (Fig. S7). Interestingly, similar patterns of environmental factors affecting *Verrucomicrobia*, *Chloroflexi*, *Chlorobi*, and *Acidobacteria* were observed, suggesting that these taxonomic groups may have adapted to similar ecological niches and carry out similar N-cycling pathways.

10.1128/mbio.03832-21.6 FIG S6 Association between the relative abundance of microbial taxa and oxygen concentration. Microbial phyla, including *Proteobacteria* and *Firmicutes*, were analyzed. Spearman’s rho was calculated for association strength. Only samples collected from the mesopelagic zone were analyzed because oxygen minimum zones were located within this zone. Download FIG S6, TIF file, 0.3 MB. Copyright © 2022 Song et al. 2022 Song et al. https://creativecommons.org/licenses/by/4.0/ This content is distributed under the terms of the Creative Commons Attribution 4.0 International license.

10.1128/mbio.03832-21.7 FIG S7 Variations explained by different geo-environmental factors for different taxonomic groups. In the left panel, the associations between the relative abundance of individual functional traits and geo-environmental factors are indicated by the heatmap, whereas the importance of geo-environmental factors in explaining the variations of individual functional traits by random forest analysis is indicated by different sizes of circles. In the right panel, variations explained by the best geo-environmental factor (the one with the largest circle) are indicated by bar plots. Download FIG S7, TIF file, 2.5 MB. Copyright © 2022 Song et al. 2022 Song et al. https://creativecommons.org/licenses/by/4.0/ This content is distributed under the terms of the Creative Commons Attribution 4.0 International license.

### Which process governs marine N cycle community assembly?

Another important question we would like to address here is which process governs N-cycling community assembly in the global ocean. Macroecologists have used species as a basic unit of community composition (52, 53). In keeping with that established metric, microbial ecologists have primarily used the operational taxonomic unit (OTU) or, more recently, the amplicon sequence variant (ASV) derived from 16S rRNA gene sequencing as a surrogate for species (54) but rarely have considered the associated functional traits. Several recent studies suggest that the environment selects for functional genes rather than species (26–28, 55). In addition, the recognized widespread functional redundancy among species in microbial communities suggests that species composition is of less importance than the functional traits they encode (25). A consensus reached recently by microbial ecologists is that both deterministic and stochastic processes structure microbial community assembly, and the question to resolve is their relative importance (54, 56). Here, considering the potential functional redundancy in marine N-cycling communities (Fig. 1A and B), we hypothesized that the composition of N-cycling functional traits was highly deterministic, while taxonomic groups were relatively more stochastic.

**FIG 4:**
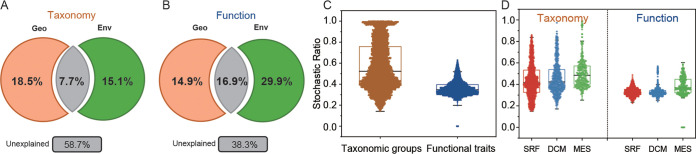
Mechanisms governing the assembly of N-cycling microbial communities. (A and B) Variation partitioning analysis of the contributions of geographic distance and environmental factors in explaining the variations of N-cycling taxonomic groups and functional traits. (C and D) Stochastic ratio representing the stochasticity of community assembly for N-cycling taxonomic groups and functional traits, as revealed by null model analyses. At both the global scale and individual layers, higher stochasticity could be observed for the community assembly of taxonomic groups than functional traits.

We first characterized the contributions of geographic distance and environmental factors (i.e., deterministic factors) in explaining the compositional variations of taxonomic groups and functional traits (Fig. 4A and B). A higher proportion of the compositional variation of functional traits could be better explained by geographic distance and environmental factors than by taxonomic group composition. For functional traits, a total of 61.9% of compositional variations could be explained by geographic distance and environmental factors, which respectively, achieved a pure explanation rate of 14.8% and 30.1% (Fig. 4A). For taxonomic groups, a total of 43.7% of compositional variations could be explained by geographic distance and environmental factors, respectively, with 18.8% and 16.3% pure explanation rates (Fig. 4B). Notably, environmental factors were mainly responsible for the compositional variations of functional traits, while geographic distance was more important in explaining the compositional variations of taxonomic groups.

Null model analysis was then employed to analyze the relative importance of deterministic and stochastic processes in structuring the N-cycling communities. Here, stochastic ratios (57, 58) were calculated for assessing the stochasticity of the composition of taxonomic groups and functional traits. Consistent with the variation partitioning analysis (VPA) results and our hypothesis, functional traits were highly deterministic. Stochastic processes only contributed 37.6% relative importance in shaping the compositional variations of functional traits. Taxonomic composition was overall relatively less deterministic such that the contribution of stochastic processes to taxonomic compositional variations was 53.9% (Fig. 4C). The same patterns were observed at individual layers (Fig. 4C and D). Such discrepant patterns between taxonomic groups and functional traits suggested that the ocean ecosystem selects for functional traits rather than taxonomic groups.

### A functional-trait-based model for microbial community assembly.

**FIG 5:**
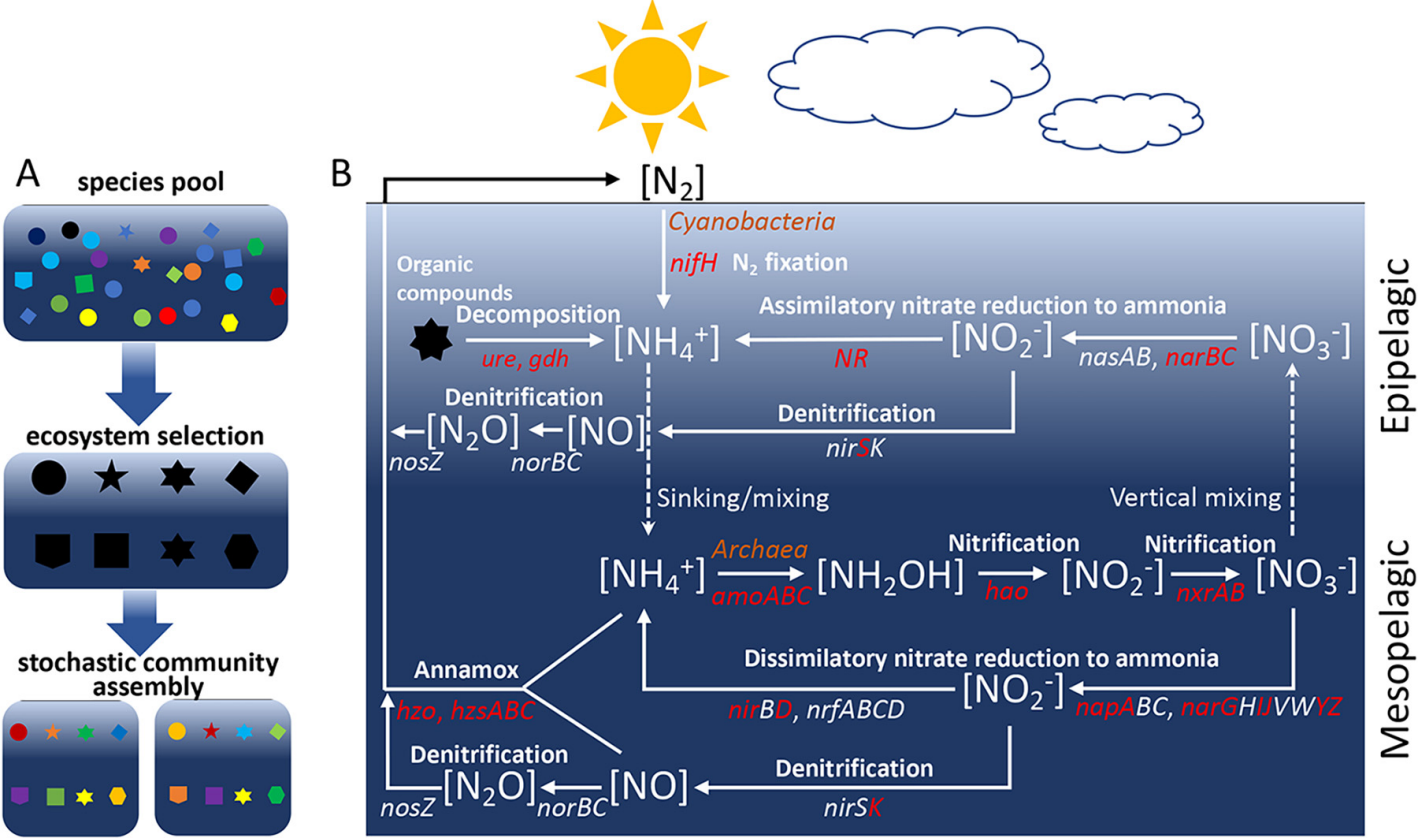
Conceptual models illustrating the community assembly and distribution of N-cycling pathways in the oceanic ecosystem. (A) A conceptual model for N-cycling community assembly. First, a regional species pool is formed, adapting to ecological niches in the ocean. Second, the ecosystem selects functional traits rather than species, unless they are highly specialized. Third, functional redundancy of microbial species leads to stochastic community assembly. In the model, different shapes represent different ecosystem functions, whereas different colors represent different microbial taxa. (B) A schematic model illustrating the distribution of N-cycling pathways in ocean. N-cycling pathways differed vertically by depth, instead of by ocean. Relative abundances of functional traits involved in N_2_ fixation, organic decomposition, and ANRA were significantly enriched in epipelagic zones, whereas those involved in nitrification, DSRN, and annamox were significantly enriched in the mesopelagic zone. Denitrification is highly detected in both epipelagic and mesopelagic zones but dominated by different functional traits. Specifically, *nirS* was significantly more abundant in epipelagic zones, while *nirK* was more abundant in the mesopelagic layer. Ammonia oxidation (NH_4_^+^ → NH_2_OH) was mainly carried out by archaea.

Integrating all of the above-described information, we propose a functional-trait-based model to explain the complex microbial community assembly (Fig. 5A). First, multiple ecological niches (e.g., epipelagic and mesopelagic zones) are generated in the ocean ecosystem by various geo-environmental factors, such as depth, temperature, and oxygen. Microorganisms that are able to survive in these ecological niches form the regional species pools. Second, in order to maintain essential ecosystem function, the environment selects microbial functional traits rather than species (55), unless the microbial species are highly specialized in particular functions, such as anaerobic or aerobic ammonia oxidation. Third, owing to functional redundancy (25), there usually exist excessive microbial taxa executing the same ecosystem function in a typical ecosystem. Such functional redundancy of microbial communities correlates positively with ecosystem stability and resilience (59). Finally, microbial taxa executing the same ecosystem functions are filtered by environmental conditions. The ones better adapted to the ecosystem are highly selected. Stochasticity is associated with this selection process, leading to varied microbial taxonomic composition but stable ecosystem function. Therefore, in addition to taxonomic groups, functional traits should be considered in analyzing microbial community assembly.

### Conclusions.

This study draws a biogeographic picture of the N-cycling microbial communities in the global ocean (Fig. 5B). The dominance of different N-cycling pathways was observed in different layers, but not in different oceanic provinces, suggesting that depth-related parameters were the major environmental factors driving the vertical variations of N-cycling pathways. Approximately 0.65% of the captured sequences encode functional traits mediating this critical biogeochemical cycle in the ocean. However, at least 57.64% of them cannot have taxonomic information assigned, mainly due to limitations of current genomic databases (60). This shortage hampers full understanding of the microbial taxa mediating the marine N cycle, especially in that new discoveries in the N cycle are still being made (8, 9, 12).

In addition to describing the biogeography and the geo-environmental drivers of the marine N cycle, this study anchored functional traits to disentangle the underlying ecological mechanism governing the complex microbial community assembly. Such attempts run counter to conventional studies that mainly rely on microbial species (54), which are usually considered the fundamental unit of selection in biology and ecology (61). Our study demonstrated an essential mechanism in ecology—the ecosystem selects microbial functional traits rather than species; functional redundancy among species comprising microbial communities, often resulting from convergent evolution and horizontal gene transfer (25, 62), not only guarantees ecosystem stability (59) but is the foundation for stochastic microbial community assembly. Therefore, we urge that functional traits be integrated into future microbial ecology studies to better clarify the mechanisms underlying community assembly, diversity-process relationships, and ecosystem responses to environmental change (63).

## MATERIALS AND METHODS

### *Tara* Oceans shotgun metagenomes and environmental factors.

The *Tara* Oceans shotgun metagenomic data sets targeting 128 samples were retrieved from the European Nucleotide Archive (ENA) under project ID ERP001736. A total of 72,492,220,288 reads were included in the data sets. To get a more representative sequence set for microbial communities driving the marine N cycle, read-based analyses rather than metagenomic assembly were carried out. To increase the accuracy of database searching, forward and reverse reads were merged into longer sequences by the program PEAR (version 0.9.11) (64). Parameters including -p 0.0001 -q 30 were applied for PEAR. An average of 264,012,963 merged reads per sample were obtained.

### Metagenomic profiling of marine N-cycling pathways.

Merged shotgun metagenome sequences were searched against the current state of the art database for N-cycling gene families, NCycDB (https://github.com/qichao1984/NCyc), a manually curated functional gene database specifically designed for profiling N-cycling pathways in shotgun metagenomes (5). The whole NCycDB (68 gene families) and a newly added gene family *nifB* were used for metagenomic profiling of N-cycling communities. To balance between speed and accuracy, the program DIAMOND (version 0.9.25) (65) was selected to search nucleotide sequences against NCycDB using the blastx mode. Parameters including -k 1 -e 0.0001 were used for DIAMOND. Functional profiles were then obtained using the perl script provided in NCycDB. Since all samples in the *Tara* Oceans project were sequenced with ultradeep sequencing depth, we performed standard normalization instead of random subsampling to the minimum sequencing depth. The total number of sequences for each sample was normalized to 100,000,000.

To obtain the taxonomic profiles for microbial communities driving the marine N cycle, sequences targeted by N-cycling gene families in NCycDB were extracted using the seqtk program (https://github.com/lh3/seqtk). Extracted sequences were then subject to taxonomic assignment by Kraken 2 (66). A local standard Kraken 2 database was built for taxonomic assignment. Taxonomic profiles were then generated for N-cycling pathways at different taxonomic levels.

### Diversity indices.

The “vegan” package in R (67) was used to calculate various diversity indices for marine N-cycling microbial communities. Specifically, the Shannon-Wiener index and Chao1 richness were calculated for within-sample diversity, i.e., alpha diversity. The Bray-Curtis dissimilarity was used to represent between-sample diversity, i.e., community dissimilarity or beta diversity. Community similarity was calculated by subtracting community dissimilarity from 1. Both within-sample and between-sample diversity indices were calculated for functional and taxonomic profiles. Principal-component analysis (PCA) was performed to explore the compositional variance among samples in different layers and oceans in a low-dimension space. The first two axes were extracted for data visualization. Multiple nonparametric statistical methods, including permutational multivariate analysis of variance (PERMANOVA), analysis of similarity (ANOSIM), and multiresponse permutation procedure (MRPP), were performed based on Bray-Curtis dissimilarities in the vegan R package (67).

### Latitudinal diversity gradient and distance decay relationship.

Two types of well-studied biogeographic patterns in ecology were analyzed, including the latitudinal diversity gradient and distance decay relationships. For the latitudinal diversity gradient, the relationship between within-sample richness and absolute latitude for the sample was analyzed. For the distance decay relationship, the relationship between log transformed community similarity and log transformed geographic distance was analyzed. Geographic distances between different samples were calculated based on the latitude and longitude coordinates using the “gdist” function in the R “Imap” package. For both latitudinal diversity gradient and distance decay relationships, linear regression analysis was carried out to calculate the slope and significance values. Analyses were done for all samples and for samples in three different layers.

### Correlating environmental factors with community diversity and composition.

To identify the potential geo-environmental factors shaping the variations of marine N-cycling microbial community diversity and composition, a total of 19 geo-environmental factors were recruited—absolute latitude, depth, temperature, oxygen, nitrates, phosphorus, NO_2_NO_3_, silicate, salinity, NO_2_, AMODIS:PAR8d, Okubo-Weiss, Lyapunov, retention, MLD, Max N_2_, Max O_2_, Min O_2_, and nitracline. Multiple statistical analyses were carried out. First, partial Mantel tests were used to evaluate the correlation between geo-environmental factors and N-cycling taxonomic and functional gene trait composition by controlling the effects of geographic distance. The Bray-Curtis dissimilarity values were used to represent the compositional variations of taxonomic and functional profiles. For distance of geo-environmental factors, the Euclidean distance was calculated. Second, linear regression analyses were conducted to investigate the relationships between each individual geo-environmental factor and within-sample and between-sample diversity indices. For within-sample diversity, the Shannon-Wiener index was used. For between-sample diversity, the first axis values of PCA were extracted. Spearman’s rank coefficient of correlation was calculated. Third, in addition to partial Mantel tests and linear regression analyses, we also employed the machine learning method random forest analysis to determine the geo-environmental factor best predicted by each taxonomic group and functional gene trait. The relative importance of geo-environmental factors in explaining the variations of taxonomic groups and functional gene traits was estimated. R packages, including vegan (67), randomForest (68), and relaimpo (69), were used for above statistical tests.

### Community assembly mechanisms.

Two different approaches were employed to investigate the potential ecological mechanisms governing the compositional variations of marine N-cycling microbial communities. First, variation partitioning analysis (VPA) was used to disentangle the relative importance of environmental and spatial factors shaping the compositional variations. For geographic variables, the principal coordinates of neighbor matrices (PCNM) procedure was used to capture all the detectable spatial scale variables based on the longitude and latitude coordinates of each sampling station (70). A forward selection procedure was then used to select spatial and environmental variables by employing a constrained analysis of the canonical correlation analysis (CCA) model. Environmental factors chosen for VPA were then determined based significance levels (*P < *0.05) until no improvement was observed when adding new variables. The explained and unexplained variation by geographic and environmental factors were determined. Second, null model analysis was performed to characterize the relative importance of deterministic factors and stochastic processes in structuring marine N cycle microbial communities. To eliminate potential influences of local and regional species richness on β diversity, null models were generated by constraining the within-sample (local) and across-sample (regional) richness (71). A total of 1,000 null models were generated, based on which, Bray-Curtis dissimilarity was calculated. An average Bray-Curtis dissimilarity matrix was then generated. Community assembly stochasticity was estimated by comparing the observed and randomized community dissimilarity, according to a modified method as described previously (54, 58). In the null model analyses, two kinds of situations were considered in stochastic strength calculation. If communities are governed by deterministic factors leading to more similar communities, the observed community similarity (*C_ij_*) between the *i*-th and *j*-th communities shall be greater than the null expectations ($\bar{E_{\mathrm{ij}}}$). If communities are governed by deterministic factors that make communities more dissimilar, the observed community similarity (*C_ij_*) between the *i*-th and *j*-th communities shall be smaller than the null expectations ($\bar{E_{\mathrm{ij}}}$). That being said, the observed dissimilarity (*D_ij_* = 1 − *C_ij_*) shall be greater than the null model dissimilarity ($\bar{G_{\mathrm{ij}}}=1\;-\;\bar{E_{\mathrm{ij}}}$). The stochastic ratio can therefore be calculated according to the following functions:

$$ST_{\mathrm{ij}}^{A}=\frac{\bar{E_{\mathrm{ij}}}}{C_{\mathrm{ij}}}\text{ if }C_{\mathrm{ij}}\;\geq \;\bar{E_{\mathrm{ij}}}$$

$$ST_{\mathrm{ij}}^{B}=\frac{\bar{G_{\mathrm{ij}}}}{D_{\mathrm{ij}}}=\frac{1\;-\;\bar{E_{ij}}}{1\;-\;C_{\mathrm{ij}}}\text{ if }C_{\mathrm{ij}}\;<\;\bar{E_{\mathrm{ij}}}$$

$$\mathrm{ST}=\frac{\sum_{\mathrm{ij}}^{n^{A}}ST_{\mathrm{ij}}^{A}\;+\;\sum_{\mathrm{ij}}^{n^{B}}ST_{\mathrm{ij}}^{B}}{n^{A}\;+\;n^{B}}$$

Both VPA and null model analyses were carried out for both taxonomic and functional profiles. R packages, including vegan (67), bioenv (72), and NST (57), were used in the analysis.
