# Supplementary figures and images for: Functional Traits Resolve Mechanisms Governing the Assembly and Distribution of Nitrogen-Cycling Microbial Communities in the Global Ocean
Source: mBio. 2022 Mar 14;13(2):e03832-21. doi: 10.1128/mbio.03832-21 (PMC9040759; doi:10.1128/mbio.03832-21)

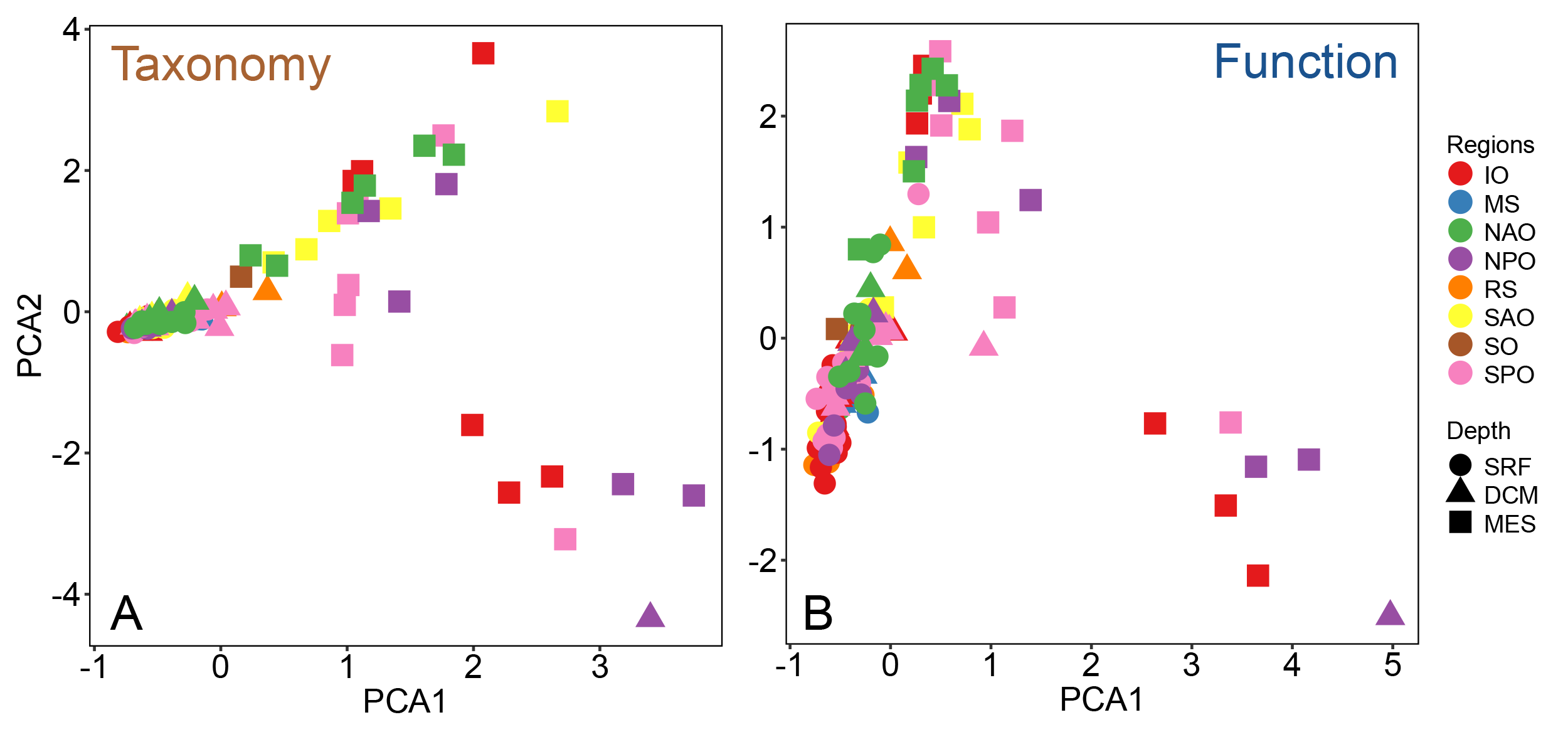

Supplement: FIG S1 [file mbio.03832-21-sf001.tif]

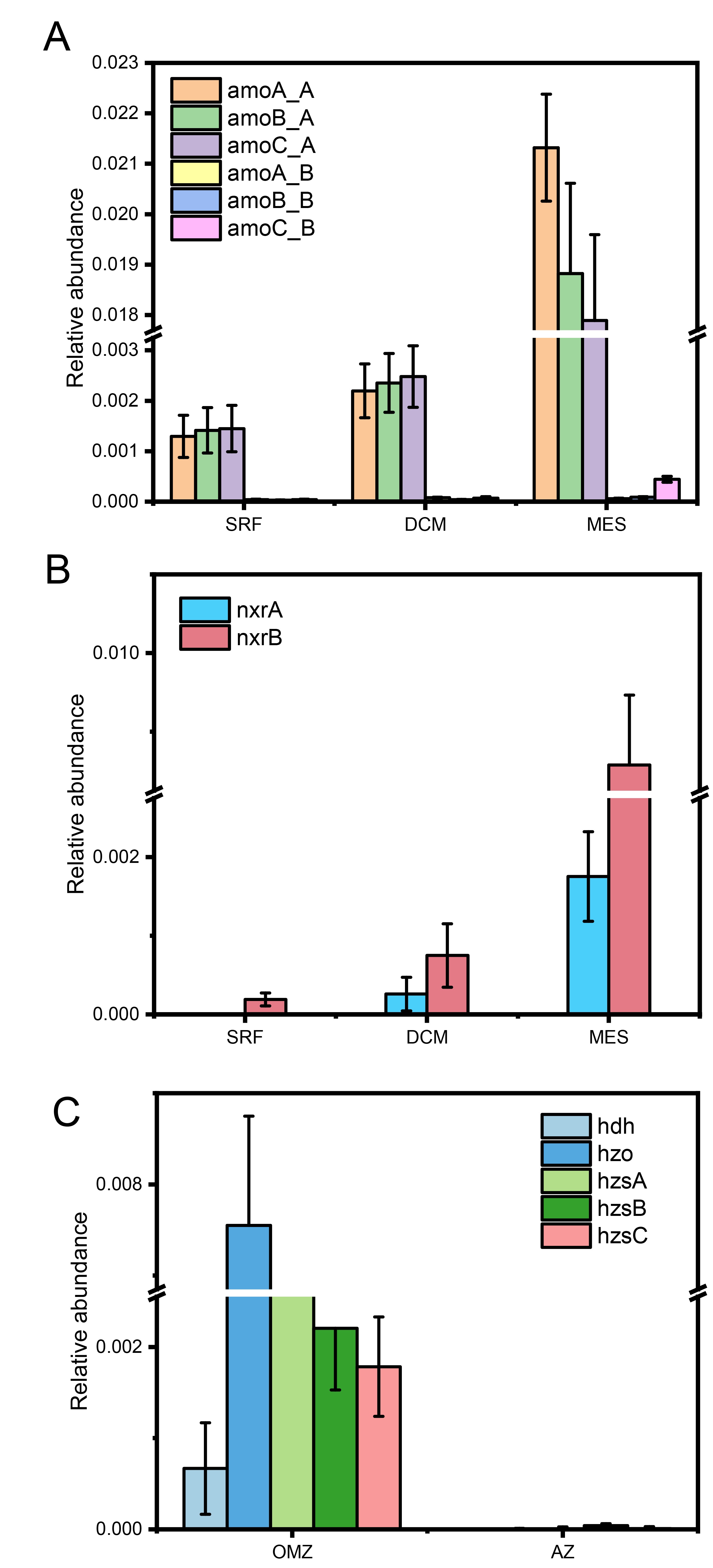

Supplement: FIG S2 [file mbio.03832-21-sf002.tif]

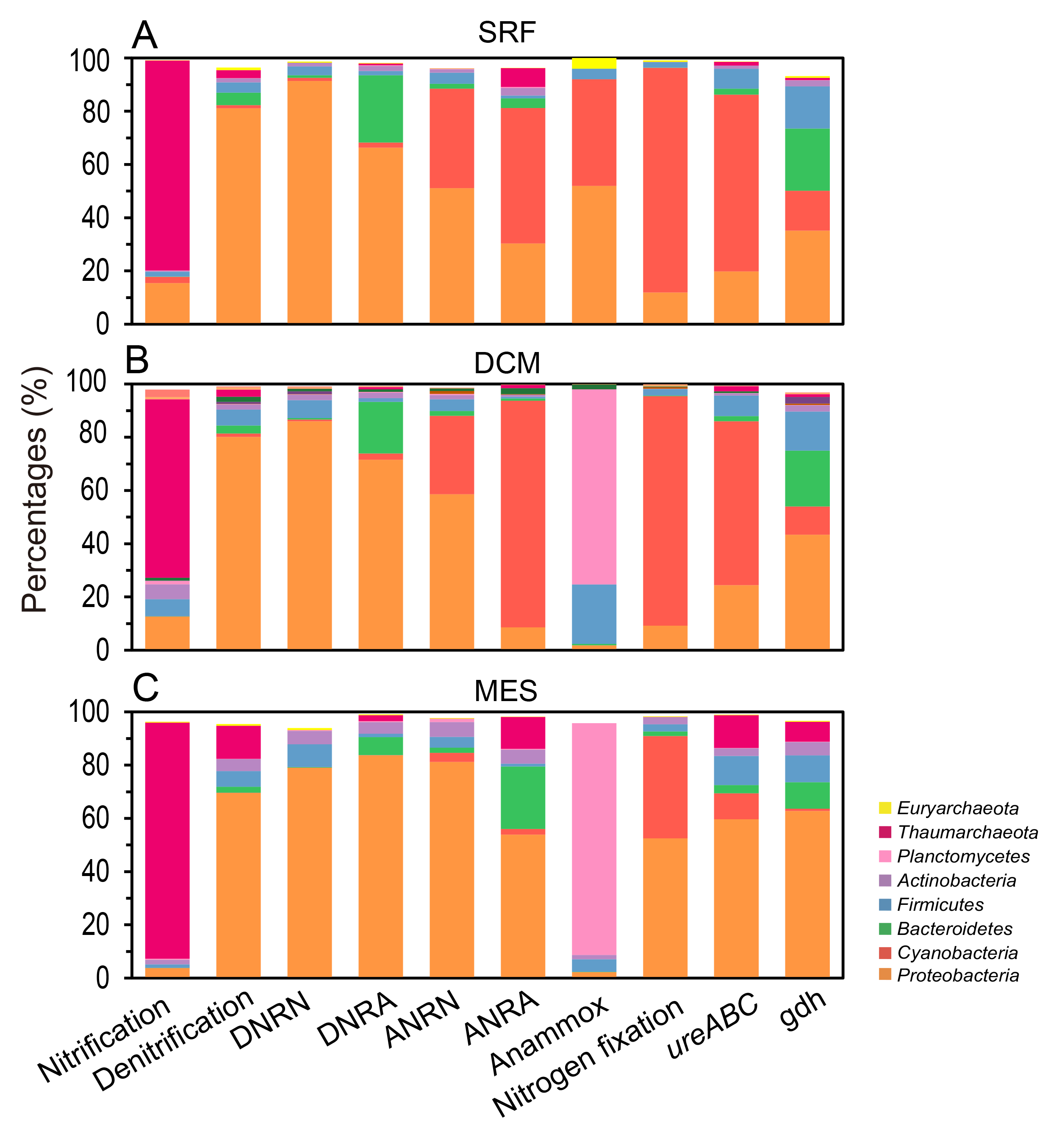

Supplement: FIG S3 [file mbio.03832-21-sf003.tif]

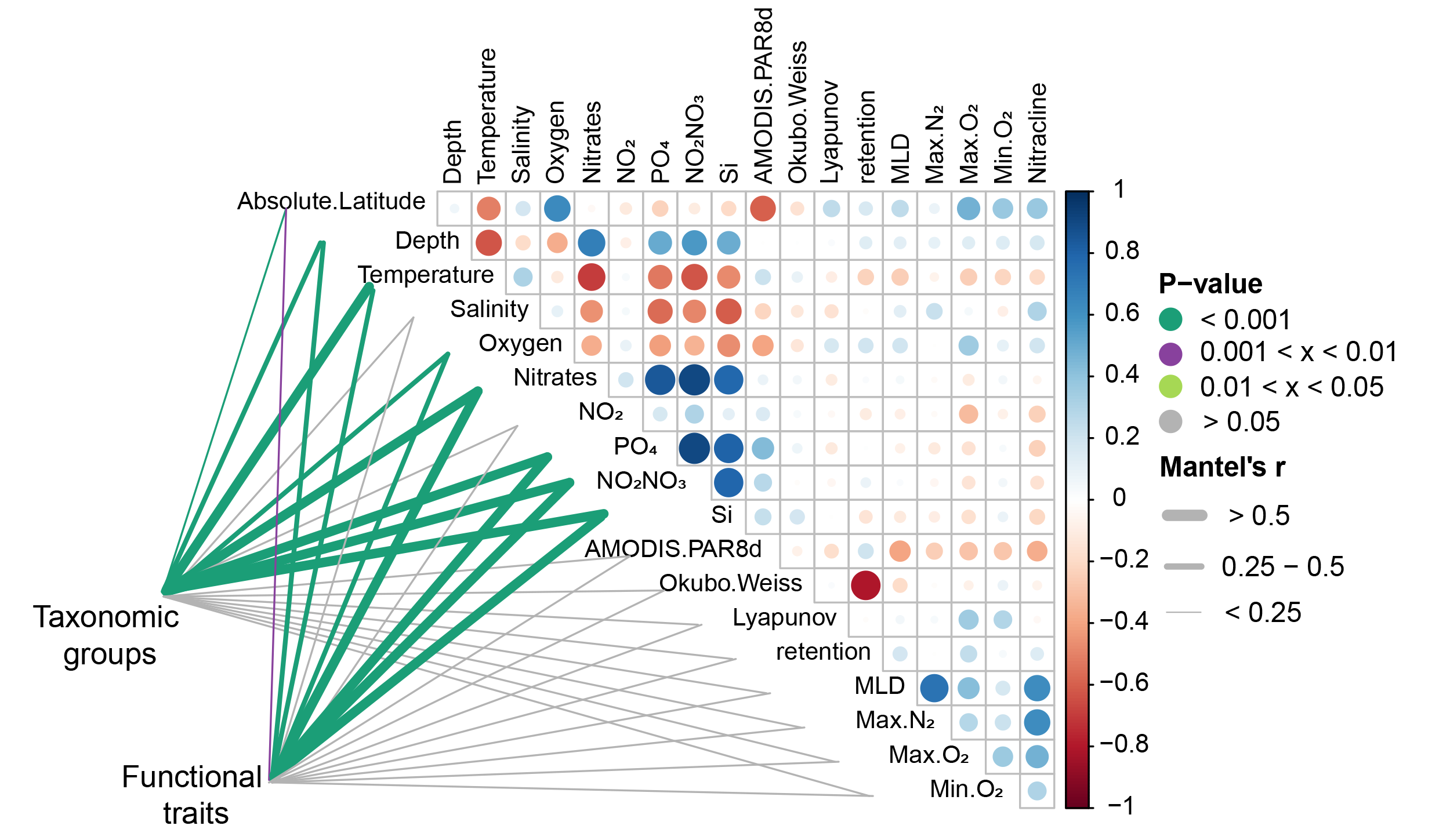

Supplement: FIG S5 [file mbio.03832-21-sf005.tif]

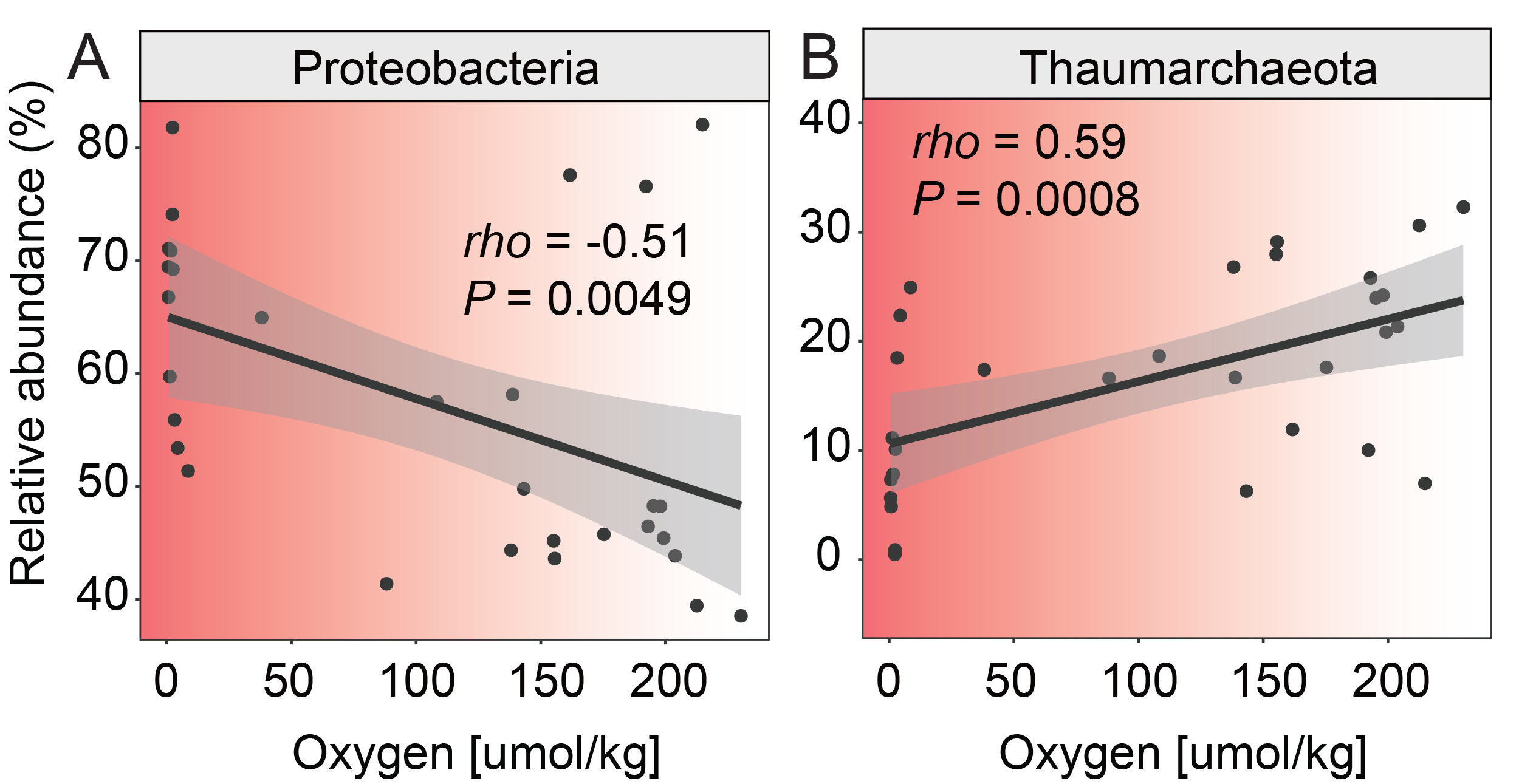

Supplement: FIG S6 [file mbio.03832-21-sf006.tif]

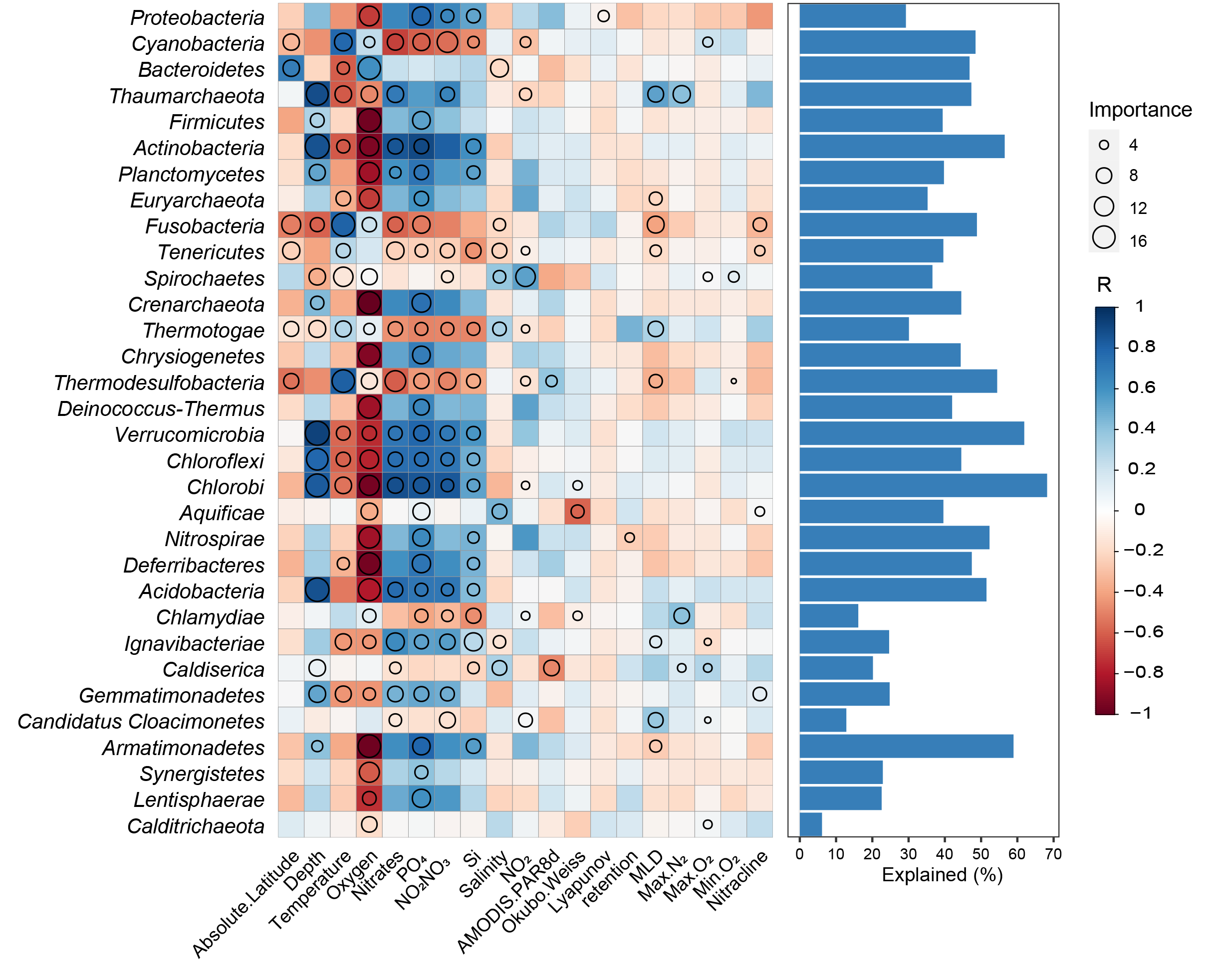

Supplement: FIG S7 [file mbio.03832-21-sf007.tif]
