# Supplementary material for: Functional Traits Resolve Mechanisms Governing the Assembly and Distribution of Nitrogen-Cycling Microbial Communities in the Global Ocean
Source: mBio. 2022 Mar 14;13(2):e03832-21. doi: 10.1128/mbio.03832-21 (PMC9040759; doi:10.1128/mbio.03832-21)
Supplement: TABLE S1 [file mbio.03832-21-st001.docx]

**Supplementary Table 1**. Statistical analysis of the compositional differences between epipelagic (MES and DCM) and mesopelagic (MES) layers. Three non-parametric statistical approaches, including permutational multivariate analysis of variance (PERMANOVA), analysis of similarity (ANOSIM), and multiple response permutation procedure (MRPP), were carried out based on Bray-Curtis dissimilarity distance matrices. Both compositions for taxonomic groups and functional traits were analyzed.

|  | PERMANOVA | | | | | ANOSIM | | | | MRPP | | | |
| --- | --- | --- | --- | --- | --- | --- | --- | --- | --- | --- | --- | --- | --- |
|  | Taxonomic | | | Functional | | Taxonomic | | Functional | | Taxonomic | | Functional | |
|  | *F* | | *P* | *F* | *P* | *R* | *P* | *R* | *P* | *δ* | *P* | *δ* | *P* |
| SRF vs DCM | | 2.678 | 0.012 | 3.709 | 0.004 | 0.038 | 0.076 | 0.034 | 0.112 | 0.584 | 0.005 | 0.249 | 0.004 |
| SRF vs MES | | 33.088 | 0.001 | 48.991 | 0.001 | 0.776 | 0.001 | 0.685 | 0.001 | 0.668 | 0.001 | 0.346 | 0.001 |
| DCM vs MES | | 24.631 | 0.001 | 30.422 | 0.001 | 0.723 | 0.001 | 0.601 | 0.001 | 0.659 | 0.001 | 0.334 | 0.001 |
